# Characterization of TROP-2 bispecific T cell engagers for immunotherapy of triple negative breast and bladder cancer

**DOI:** 10.3389/fimmu.2026.1794705

**Published:** 2026-03-12

**Authors:** Carlos Ávila-Nieto, Gundram Jung, Helmut R. Salih, Ilona Hagelstein

**Affiliations:** 1 German Cancer Consortium (DKTK), Partner Site Tuebingen, a Partnership Between German Cancer Research Center (DKFZ) and University Hospital Tübingen, Tübingen, Germany; 2 Clinical Collaboration Unit Translational Immunology, Department of Internal Medicine, University Hospital Tübingen, Tübingen, Germany; 3 Cluster of Excellence iFIT (EXC 2180) “Image-Guided and Functionally Instructed Tumor Therapies”, Eberhard Karls University of Tübingen, Tübingen, Germany

**Keywords:** immunotherapy, triple negative breast cancer, bladder cancer, TROP-2, CD3, bispecific antibody

## Abstract

T cell-based immunotherapy has markedly expanded the therapeutic options in numerous cancers. However, these approaches still achieve only limited clinical benefit in triple negative breast cancer (TNBC) and bladder cancer. Although immune checkpoint inhibitors improve outcomes for a subset of patients, no T cell-redirecting therapies such as CAR-T cells or bispecific antibodies (bsAbs) have been approved for either indication. Trophoblast cell surface antigen 2 (TROP-2) is highly expressed across several epithelial cancers including TNBC and bladder cancer, but has been primarily exploited as a target for antibody drug conjugates (ADCs) with limited exploration in T cell-engaging constructs. Here, we report on the generation and characterization of a panel of TROP-2×CD3 bsAbs containing clinically validated TROP-2 binders and CD3 binders with distinct affinities. All bsAbs induced robust T cell activation, cytokine secretion and sustained T cell expansion, resulting in potent T cell-mediated cytotoxicity against TNBC and bladder cancer cells with either high or low levels of TROP-2 expression. Notably, combining a TROP-2 binder with enhanced tumor selectivity and a low-affinity CD3 binder increased discrimination between high and very low TROP-2-expressing cells while (reducing cytokine release without compromising anti-tumor efficacy. Thus, TROP-2-directed bsAbs can achieve effective tumor cell killing without over dependence on antigen density, in contrast to ADC-based approaches. Our results support further development of TROP-2×CD3 bsAbs as immunotherapy for solid tumors with heterogeneous or low TROP-2 expression.

## 1 Introduction

Over the recent years, immunotherapy has revolutionized oncological treatment. Among the most impactful developments are T cell-mobilizing strategies, which have demonstrated meaningful survival benefits across multiple malignancies (1–3). These include immune checkpoint inhibitors (ICIs), chimeric antigen receptor (CAR)-T cells and bispecific antibodies (bsAbs). Both CAR-T cells and bsAbs, promote T cell activity through recognition of tumor-associated antigens (TAAs) expressed on malignant cells. Although recent clinical approvals of bsAbs are encouraging (4, 5), so far bsAbs and CAR-T cells have shown only limited success in solid tumors as compared with their remarkable efficacy in lymphoid malignancies (6, 7).

Trophoblast cell surface antigen 2 (TROP-2), also known as tumor-associated calcium signal transducer 2 (TACSTD2), is a type 1 transmembrane glycoprotein involved in multiple intracellular signaling pathways related to cell proliferation, migration and invasion (8). Initially identified in trophoblasts and exhibiting limited expression in normal tissues, TROP-2 is aberrantly overexpressed in a broad spectrum of solid tumors, including breast, gastric, colon, pancreas, lung, urinary bladder and ovarian cancer (9, 10). Elevated TROP-2 expression has further been associated with metastasis and poor prognosis in several malignancies, such as breast and pancreatic cancer (11–13). Its clinical relevance is supported by the success of TROP-2-directed antibody drug conjugates (ADCs). Two such ADCs - sacituzimab govitecan (SG) and datopotamab deruxtecan (Dato-DXd) - have received FDA approval for the treatment of triple-negative breast cancer (TNBC) and HR+/HER2- breast cancer respectively, and Dato-DXd, also received FDA accelerated approval for non-small cell lung cancer (14–16). These features identify TROP-2 as an attractive therapeutic target to be explored in the development of bsAbs.

In this work, we constructed TROP-2×CD3 bsAbs using two distinct TROP-2 binders, clone RS7 derived from SG and clone TINA1 from Dato-DXd. Each was paired with two CD3-binding scFvs with differing affinity for CD3. The functionality of the different TROP-2×CD3 bsAbs to mediate T cell-dependent antitumor activity was then evaluated in TNBC and bladder cancer models. Our findings demonstrate the suitability of TROP-2 as target for bsAb-based immunotherapy and support continued development for solid tumors.

## 2 Materials and methods

### 2.1 Relative gene and protein expression of TROP-2 based on TCGA and CPTAC database analysis

Data on the relative TROP-2 mRNA expression in bladder and breast tumor tissues and normal tissues was obtained from the Cancer Genome Atlas (TCGA) database and the GTEx projects, and analyzed and plotted using the Gene Expression Profiling Interactive Analysis (GEPIA) and the University of ALabama at Birmingham CANcer data analysis Portal (UALCAN). Data on the relative TROP-2 protein expression was obtained from the Clinical Proteomic Tumor Analysis Consortium (CPTAC), and evaluated and plotted from the University of ALabama at Birmingham CANcer data analysis Portal (UALCAN).

### 2.2 BsAbs production and purification

The variable domains of SG (RS7 clone), Dato-DXd (TINA1 clone), and MOPC-21 (used as control) were inserted into a human Igγ1κ backbone-based IgGsc molecule with C-terminal scFvs (17). The constructs were produced in ExpiCho cells (Gibco, Carlsbad, CA, USA) and purified from culture supernatant by affinity chromatography on Mabselect affinity columns (GE Healthcare, Munich, Germany) followed by analytical and preparative size exclusion chromatography using Superdex S200 Increase 10/300GL and HiLoad 16/60 columns (GE Healthcare). Integrity and purity of bsAbs were analyzed by sodium dodecyl sulfate polyacrylamide gel electrophoresis (SDS-PAGE) (ThermoFischer Scientific, Waltham, MA) and Coomassie G-250 staining (Carl Roth GmbH & Co. KG, Karlsruhe, Germany) under reduced and non-reduced condition. Endotoxin levels were evaluated with EndoZyme II (BioMerieux, Marcy-l’Étoile, France) according to the manufacturer’s instructions and < 0.5 EU/ml.

### 2.3 Cells

Bladder cancer cell lines (RT4 and T-24), TNBC cell lines (HCC-70 and MDA-MB-231) and PANC-1 and MCF10A cell line were obtained from ATCC (American Type Culture Collection). Cells were routinely screened for mycoplasma contamination every three months. Cell identity was periodically confirmed by verifying the immunophenotype reported by the supplier using flow cytometry. Peripheral blood mononuclear cells (PBMCs) of healthy donors were obtained after informed consent and isolated by density gradient centrifugation (Biocoll; Biochrom, Berlin, Germany). PBMCs were viably frozen and stored in liquid nitrogen.

### 2.4 BsAbs binding characterization and *in vitro* activity

To assess bsAb-dependent effects on cell viability and metabolic activity, 10, 000 RT4, MDA-MB-231, MCF-10A, or PANC1 cells were seeded per well and cultured for 48 h in the presence or absence of the indicated constructs (2 nM). Subseqently, WST reagent (Roche, Basel, Switzerland) or the CellTiter-Glo^®^ Luminescent Cell Viability Assay (Promega, Madison, WI) was added, and viability/metabolic activity was determined according to the manufacturers’ instructions. BsAb-dependent effects on cell migration was evaluated by wound scratch assay. The same cell lines were grown to confluence in Incucyte^®^ Imagelock 96-well Microplates (Sartorius, Göttingen, Germany). A uniform scratch was created in the cell monolayer using Incucyte^®^ 96-Well Woundmaker Tool (Sartorius), and cells were washed with PBS to remove debris. Cells were then incubated with the indicated constructs (10 nM) in complete medium. Wound closure was monitored every 3 h for 24–72 h using the IncuCyte live-cell analysis system, and the relative wound area was quantified with the IncuCyte Wound-Scratch analysis software.

Binding of primary Ab was detected by PE-conjugated donkey anti-human IgG or goat anti-mouse IgG reagents (Jackson ImmunoResearch, West Grove, USA). Flow cytometry was performed using the BD FACSCanto II and BD LSRFortessa devices. GraphPad Prism 9 (GraphPad Software) was used to calculate EC_50_ values. T cell activation and target cell lysis were evaluated using flow cytometry-based assays. PBMCs were incubated with RT4 or MDA-MB-231 cells at the indicated effector:target (E:T) ratio in the presence or absence of bsAbs dose titration. T cells were screened using fluorescent dye-conjugated Abs directed to CD4, CD8a, CD45RO, CD62L, CD69 and CD25 (BioLegend, San Diego, CA), and the target cells were identified using anti-EpCam Ab (BioLegend) or CellTraceTM Violet (ThermoFischer Scientific). Absolute cell numbers were assessed using equal numbers of BD CompBead (BD Biosciences, San Diego, CA). 7-AAD (BioLegend) staining was used to discriminate from live- and dead-cell.

For quantification of cytokines, supernatants were collected after 8 h. IFN-γ secretion was measured by ELISA. ELISA plates were coated with IFN-γ monoclonal antibody (clone 2G1, ThermoFischer Scientific). After blocking, standard and cell supernatant were added and incubated overnight (4 °C). Commercial rh IFN-γ (ImmunoTools GmbH, Friesoythe, Germany) was used as standard and prepared in RPMI cell medium. After that, plates were washed and incubated for 1h with Biotin-conjugated IFN-γ monoclonal antibody (clone B133.5, ThermoFischer Scientific). Then, plates were washed and incubate 1h with Streptavidin-horseradish peroxidase conjugate (ThermoFischer Scientific). Plates were developed by adding TMB substrate solution (SeraCare, Milford, MA) and stopped using 1M H_3_PO_4_. The signal was analyzed as the optical density (OD) at 450 nm. The blank OD was subtracted from each sample measurement. Secretion levels of IL-2, TNF and IL-6 were quantified using LEGENDplex™ bead-based multiplex assays (BioLegend), in accordance with the manufacturer’s instructions.

### 2.5 T cell proliferation and T cell memory subset analysis

Healthy PBMCs were incubated for 6 days with tumor cells at the indicated E:T ratio and in presence or absence of bsAbs at 1nM. On day 3, fresh tumor cells and new bsAbs preparation were added to the plate. On day 6, cells were collected and T cell proliferation and memory subset formation was analyzed by flow cytometry based on CD4, CD8a, CD45RO and CD62L expression.

### 2.6 Long-term killing assay

PBMCs from healthy donors were co-cultured with tumor cells at an E:T ratio of 5:1, either in the presence or absence of bsAbs or control at 1 nM. Cytotoxicity was monitored in real time using the xCELLigence RTCA system (Roche Applied Science, Penzberg, Germany).

### 2.7 Statistics

Values are presented as mean ± standard error of the mean (SEM). Significant differences were calculated using one-way ANOVA with Bonferroni correction for multiple comparison. P values are indicated as follows: *p < 0.05, **p < 0.01, ***p < 0.001, ****p < 0.0001. Statistical analyses were conducted using GraphPad Prism 9 (GraphPad Software).

## 3 Results

### 3.1 Evaluation of TROP-2 expression in TNBC and bladder cancer

**Figure 1:**
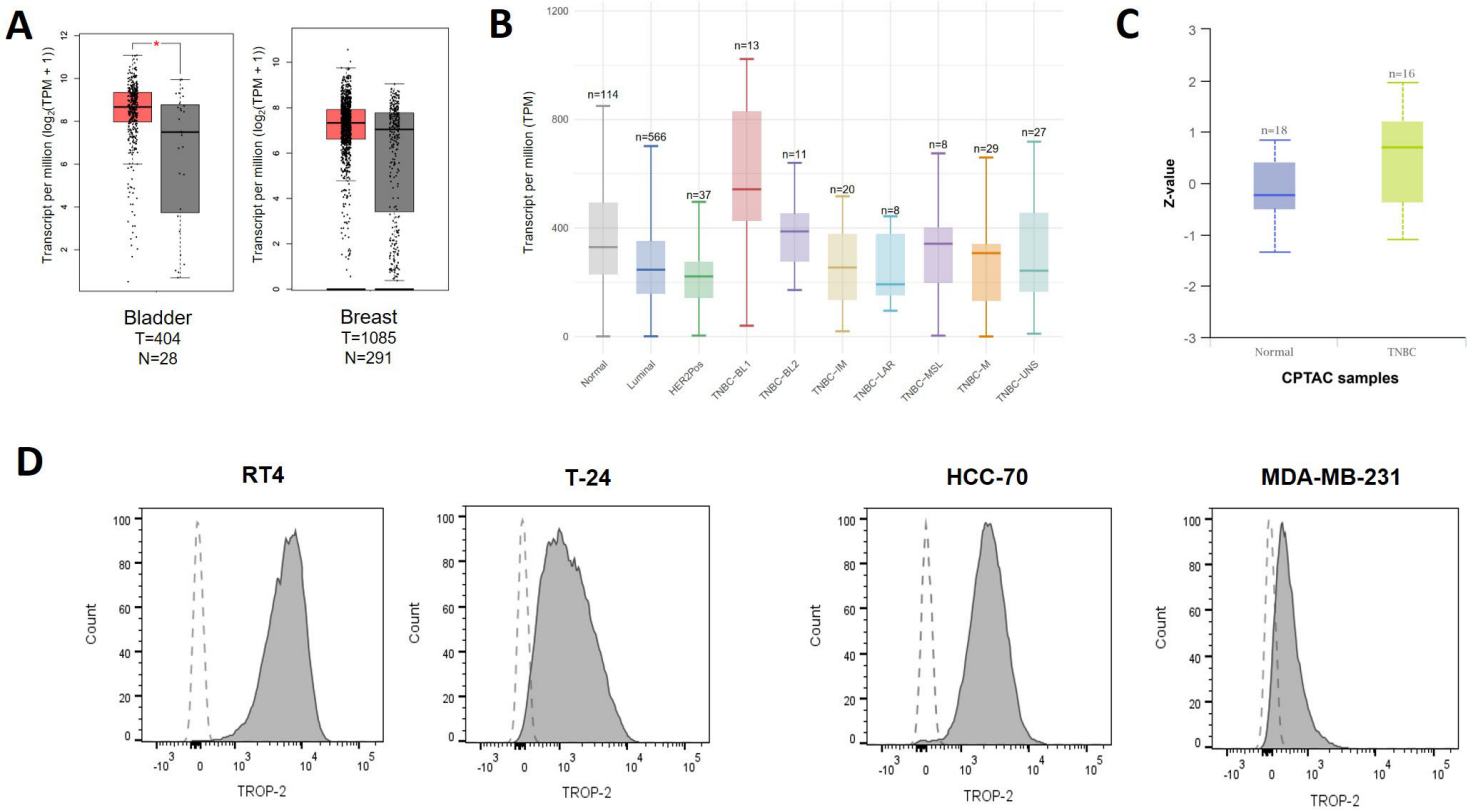
TROP-2 expression in TNBC and bladder cancer. **(A)** TROP-2 mRNA expression levels from TCGA datasets were analyzed in the indicated cancer types using the GEPIA online tool. **(B)** TROP-2 mRNA expression levels from TCGA were assessed across different breast cancer subtypes using the UALCAN portal. **(C)** TROP-2 protein expression data from the CPTAC database were evaluated in TNBC subtype using the UALCAN online tool. **(D)** TROP-2 expression levels on bladder cancer (RT4 and T-24) and TNBC cell lines (HCC70 and MDA-MB-231) were assessed by flow cytometry (shaded area, anti-TROP-2; blank area, control isotype).

As a first step we analyzed TROP-2 mRNA expression for the target indications TNBC and bladder cancer using TCGA datasets. TROP-2 expression levels were significantly elevated in bladder cancer tissue samples (n=404) compared with normal bladder tissues (n=28), whereas breast cancer (n=1085) showed only a modest increase relative to normal breast tissue samples (n=291) (**Figure 1A**). Stratification of breast tumors by molecular subtype revealed that both TROP-2 mRNA and protein expression were highest in TNBC, consistent with previous reports (18, 19) (**Figures 1B, C**).

Next, we assessed surface TROP-2 expression in bladder and TNBC cell lines by flow cytometry. All tested cell lines displayed varying TROP-2 expression (**Figure 1D**). Among them, RT4 and MDA-MB-231 showed the highest and the lowest TROP-2 levels, respectively (**Figure 1D**, **Supplementary Figure 1A**), and were therefore selected as representative high- and low- expression cell models for subsequent bsAb characterization.

### 3.2 Generation and characterization of TROP-2×CD3 bsAbs in TNBC and bladder cancer cell lines and T cell

**Figure 2:**
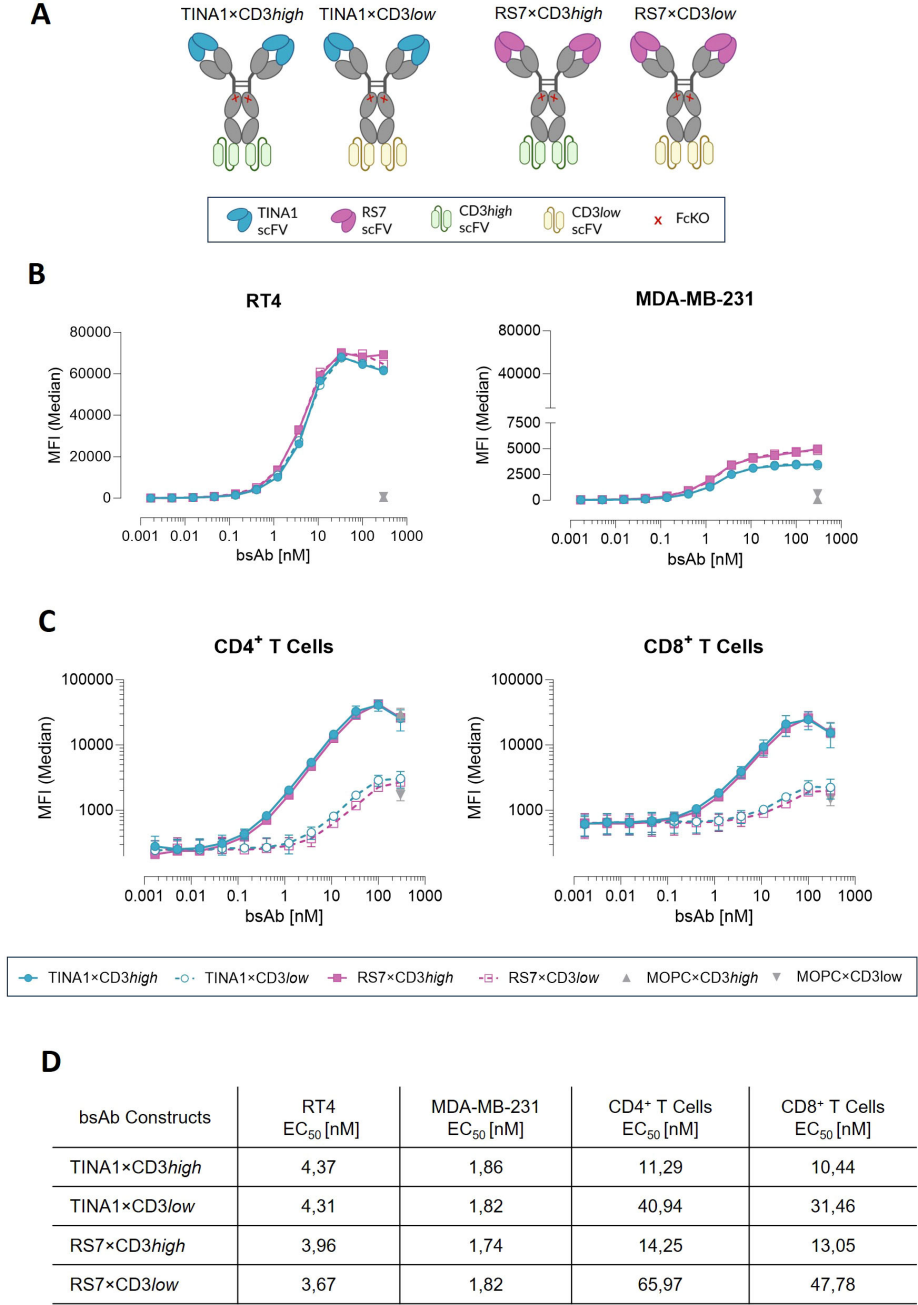
Characterization of TROP-2×CD3 bsAb constructs with target cells and effector cells. **(A)** Overview of the TROP-2 bsAbs panel with distinct anti-TROP-2 and anti-CD3 scFVs. Created with biorender.com. **(B)** Binding titration of TROP-2×CD3 bsAbs to RT4 and MDA-MB-231 cell lines by flow cytometry. **(C)** Binding titration of TROP-2×CD3 bsAbs to CD4^+^ and CD8^+^ T cells from healthy donor PBMCs (n=2) analyzed by flow cytometry (filled circles: TINA1×CD3high; open circles: TINA1×CD3low; filled squares: RS7×CD3high, and open squares: RS7×CD3low). **(D)** EC_50_ values obtained from binding experiments shown in panels **(B, C)**. Mean ± standard error of the means (SEM) is shown.

Next, we cloned the variable domains of the TROP-2 binding parts of SG (clone RS7) and Dato-DXd (clone TINA1) into our previously described IgGsc-based bsAb format (17). In addition, the single-chain sequences of UCHT-1 with high CD3-affinity (CD3high) or a UCHT-1 variant harboring four CDR-H2 mutations and one FR-H3 mutation to reduce CD3 affinity (CD3low) were incorporated as effector arms at the C-terminus of the bsAb backbone (20) (**Figure 2A**). All four constructs were then produced and the expected size and purity was analyzed by SDS-PAGE and Coomassie G-250 staining (**Supplementary Figure 2A**). Binding titration of the different TROP-2 bsAbs using the selected RT4 and MDA-MB-231 cell lines yielded EC_50_ values of ~4, 1 nM and ~1, 81 nM, respectively (**Figure 2B**). No substantial differences were observed between TINA1- and RS7-based constructs, regardless of TROP-2 expression levels (**Figure 2D**), and no-specific binding was detected in the TROP-2 negative cell line PANC-1 (**Supplementary Figures 3A, B**). Because TROP-2 has been exploited as an ADC target amongst others due to its ability to internalize (21, 22), we evaluated how receptor internalization would impair TROP-2 bsAbs binding. After 24-hour pre-incubation with maximal bsAbs concentrations, only a modest reduction in subsequent binding was detected at the highest dose (**Supplementary Figure 1B**). Next, we measured binding of our TROP-2 bsAbs to T cells. As expected, CD3high constructs showed lower binding EC_50_ values (~12 nM) than CD3low constructs (EC_50_ values ~46, 5 nM) and bound comparably to CD4^+^ and CD8^+^ T cells irrespective of the contained TROP-2 binder (**Figures 2C, D**).

Given that TROP-2 expression has been associated with enhanced proliferation and migration in cancer cells, we investigated whether binding of our TROP-2 bsAbs could modulate TROP-2 signaling in the absence of lymphocytes. Cell viability analyses using CellTiter-Glo and WST assays revealed no significant differences between untreated cells and those incubated with the bsAbs (**Supplementary Figures 1C, D**). Consistently, wound healing assays showed no differences in migratory capacity in either RT4 or MDA-MB-231 cells upon bsAb treatment (**Supplementary Figure 1E**).

### 3.3 Evaluation of T cell activation and anti-tumor activity

**Figure 3:**
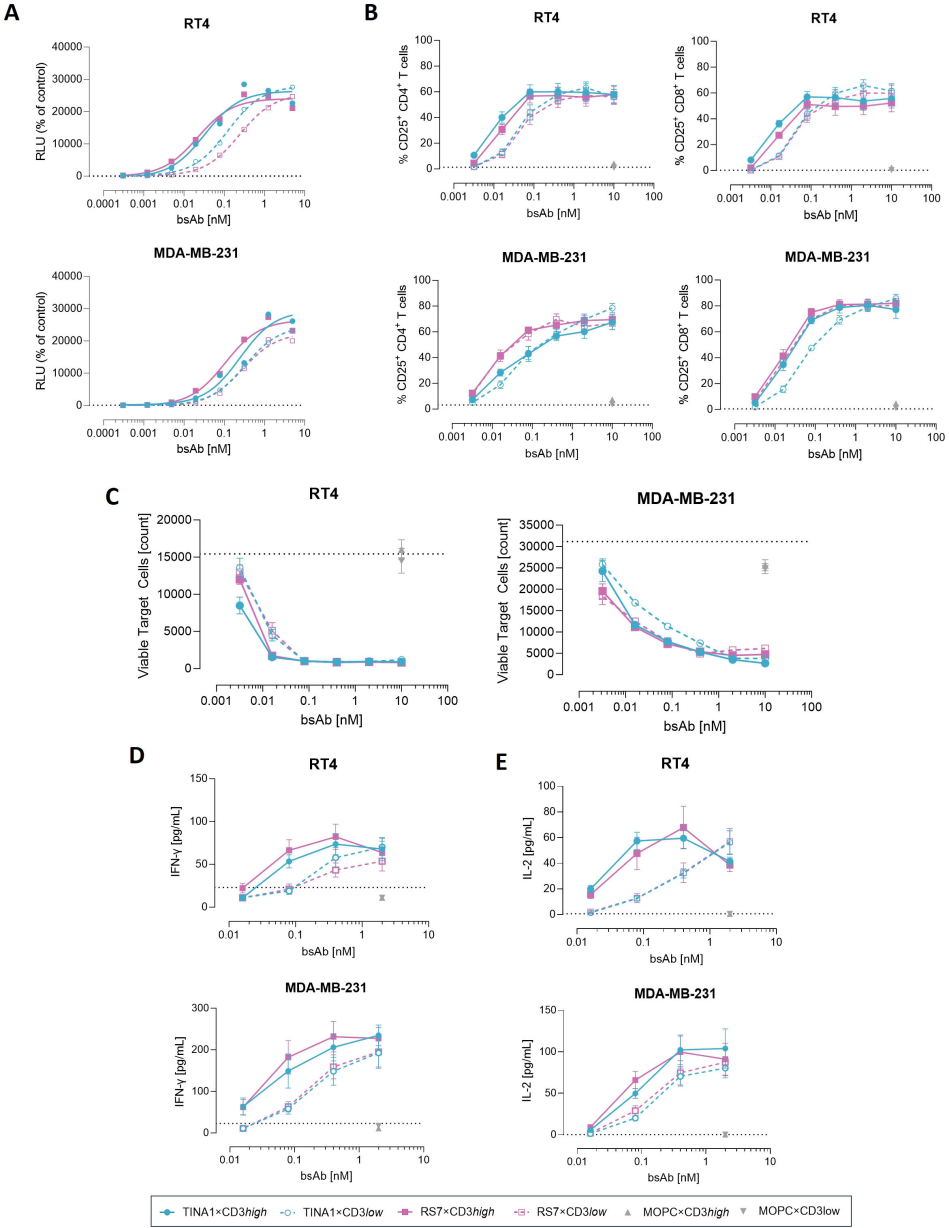
T cell activation and tumor cell cytotoxicity mediated by TROP-2×CD3 bsAbs. **(A)** RT4 and MDA-MB-231 cells were incubated with TCR/CD3 effector cells in the presence or absence of serial dilutions of TROP-2 bsAbs. Luminescence was measured after 6 h **(B)** The indicated cell lines were co-cultured with PBMCs (n=4) in the presence of the indicated bsAbs at an E:T ratio of 2:1. CD4^+^ and CD8^+^ T cell activation was analyzed by flow cytometry based on the expression of CD25 after 72h of co-culture. **(C)** Tumor cell cytotoxicity was measured by flow cytometry after 72h and is shown as the number of viable tumor cells. **(D)** Levels of IFN-γ were analyzed by ELISA in supernatants after 8h incubation. **(E)** IL-2 levels in in supernatants after 8h were quantified by LEGENDplex. Mean ± standard error of the mean (SEM) is shown.

To comparatively assess CD3-mediated T cell activation by the various TROP-2 bsAbs, we performed a T cell activation reporter assay using CD3^+^ effector cells in co-culture with the selected cancer cell lines. All TROP-2 bsAbs induced TCR/CD3 signaling in a concentration-dependent manner. Across both target cell types, CD3high variants exhibited lower EC_50_ values for T cell activation than their CD3low counterparts. Activation EC_50_ values were rather comparable between RS7- and TINA1-based constructs, even when RS7×CD3low demonstrated a slightly higher EC_50_ compared with TINA1×CD3low when using RT4 cells (0.24 nM vs. 0.12 nM) (**Figure 3A**). T cell activation was further confirmed by CD25 upregulation measured by flow cytometry after three days of co-culture of PBMC with tumor cells. Maximal CD25 induction for both CD4^+^ and CD8^+^ T cells was achieved by TROP-2 bsAb concentrations as low as 0.1 nM, with only minor differences between the two TROP-2 binders or CD3high- and CD3low-variants at higher doses. Subtle differences in activation levels emerged at lower bsAbs concentrations. Isotype controls (MOPC×CD3high and MOPC×CD3low) did not induce T cell activation at the maximal concentration and with only minimal activation observed in the TROP-2-negative PANC-1 cell line, confirming the strict target-depending activity of all TROP-2-directed constructs (**Figure 3B**, **Supplementary Figure 3C**). Next, we evaluated the ability of the constructs to induce cytolytic activity. Robust anti-tumor activity was observed against both cancer cells lines, with tumor viability being markedly reduced compared to untreated, isotype control conditions and TROP-2 negative cell line (**Figure 3C**, **Supplementary Figure 3D**). For RT4 cells, maximal cytotoxicity was retained at concentrations as low as 0.1 nM, whereas with MDA-MB-231 cells a more pronounced concentration-dependent decline in efficacy was observed, consistent with their lower TROP-2 expression. In line with T cell activation results, differences in tumor cell lysis between CD3high- and CD3low- constructs were most pronounced at low bsAb concentrations, while comparable cytotoxicity was observed at higher doses. Binder-specific effects emerged predominantly in the low-TROP-2 MDA-MB-231 model: RS7×CD3low maintained activity comparable to RS7×CD3high across all concentrations, whereas TINA1×CD3low showed reduced efficacy at low concentrations as compared to its CD3high counterpart. With RT4 cells, no substantial differences were detected between RS7- and TINA1-based bsAbs (**Figure 3C**). Consistent with the cytotoxicity data, IFN-γ and IL-2 levels in co-culture supernatants increased in a dose-dependent manner after 8 hours and were absent with the isotype controls or when target TROP-2 negative tumor cell (**Figures 3D, E**, **Supplementary Figure 3E**). As expected, the CD3low configuration resulted in reduced IFN-γ and IL-2 secretion compared to CD3high variants, with similar patterns observed for both TROP-2 binders. TNF and IL-6 secretion did not differ significantly between conditions (**Supplementary Figures 4A, B**).

Despite TROP-2 expression in normal tissues is restricted, some epithelial compartments can display detectable levels. To evaluate potential on-target, off-tumor effects of our TROP-2 bsAbs, we included the non-tumorigenic epithelial cell line MCF10A in our assays. MCF10A cells exhibited TROP-2 binding levels similar to MDA-MB-231, despite slightly lower surface expression (**Supplementary Figures 5A–C**). After 72 h of co-culture, CD4^+^ and CD8^+^ T cells were activated, but maximal activation and activation dynamics at decreasing concentrations were lower in MCF10A compared to MDA-MB-231 and RT4 (**Supplementary Figure 5D**). Cytotoxicity was observed at higher bsAb concentrations but decreased rapidly at lower doses and remained substantially lower than in MDA-MB-231 and RT4 cells (**Supplementary Figures 5E, G**). Notably, TINA1×CD3low exhibited the highest cytotoxic EC_50_ in MCF10A (0.5021 nM), which resulted in the greatest selectivity index when comparing cells with high (RT4), low (MDA-MB-231), and very low (MCF10A) TROP-2 expression. All constructs showed a selectivity index >10 when comparing between low and very low TROP-2 levels. Consistent with these findings, IFN-γ secretion was lower in MCF10A and detectable only at the highest bsAb concentrations. The CD3low constructs induced minimal IFN-γ release in MCF10A after 8 h of co-culture, in contrast to the robust response seen in MDA-MB-231 and RT4 cells (**Supplementary Figure 5F**).

### 3.4 T cell expansion and sustained effector function

**Figure 4:**
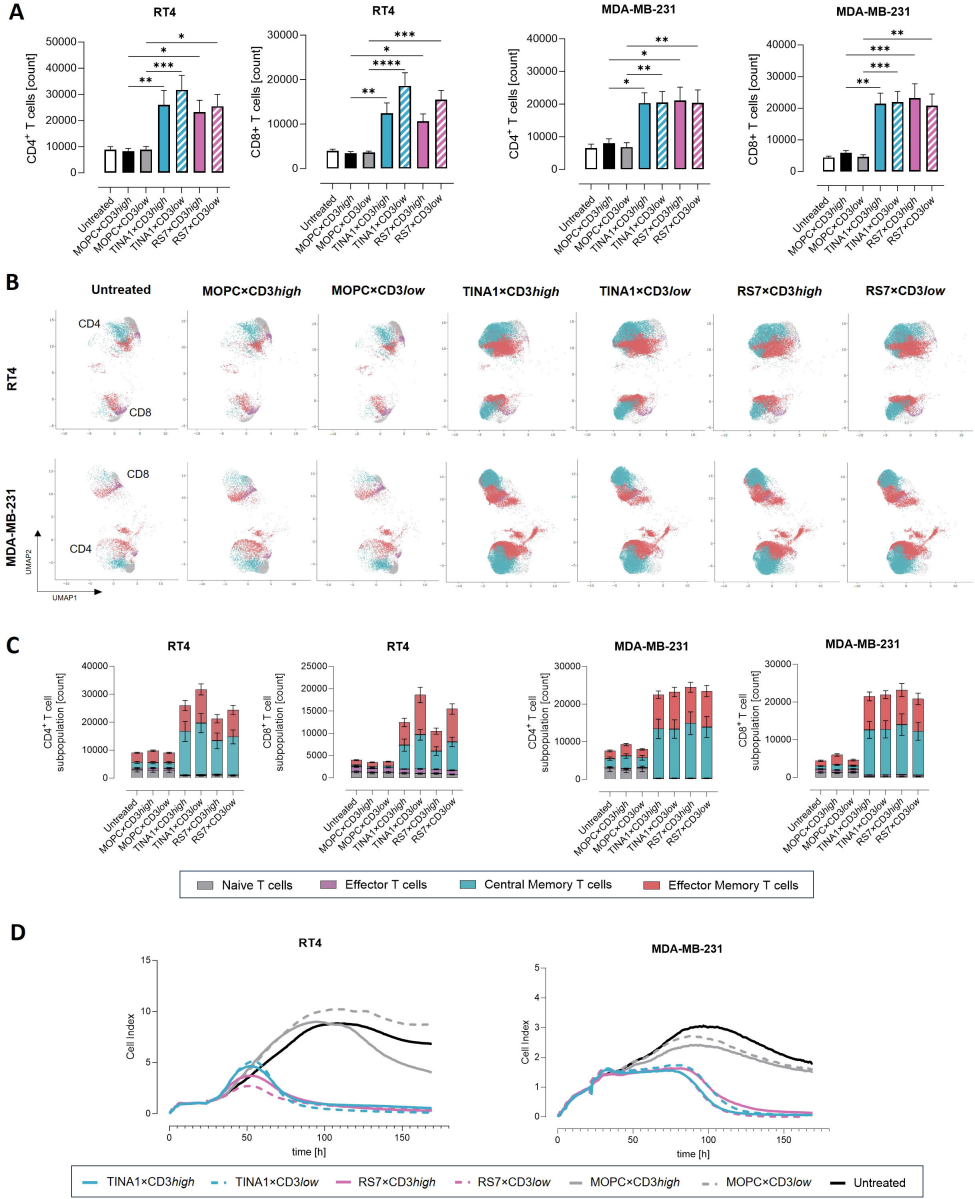
T cell expansion and sustained effector function. RT4 and MDA-MB-231 cell lines were cocultured with PBMCs (n=4) and 1nM of the indicated bsAbs for 6 days at an E:T ratio of 2:1. Tumor cells and bsAbs were renewed on day 3. **(A)** On day 6, flow cytometry was used to analyze CD4^+^ and CD8^+^ T cell counts. **(B, C)** CD4^+^ and CD8^+^ T cell subsets were evaluated by flow cytometry on day 6. Effector (CD62L^−^CD45RO^−^), naïve (CD62L^^+^^CD45RO^−^), effector memory (CD62L^−^CD45RO^^+^^) and central memory (CD62L^^+^^CD45RO^^+^^) T cell subsets were defined based on CD62L and CD45RO expression **(B)** Representative Uniform Manifold Approximation and Projection (UMAP) analyses were performed with Cytolution (https://cytolytics.de). **(C)** Bar graphs show the pooled data for T cell subsets. **(D)** Long-term cytotoxic effects of PBMCs (n=4) against RT4 and MDA-MB-231 in the presence or absence of indicated bsAbs (1nM) were analyzed using the xCELLigence system. Mean ± standard error of the mean (SEM) is shown. Statistical significance was calculated using one-way ANOVA with Bonferroni correction for multiple comparisons. *p < 0.05, **p < 0.01, ***p < 0.001, ****p < 0.0001.

Since T cell expansion is essential to combat high tumor burden, we assessed T cell proliferation and memory subset distribution by flow cytometry after 6 days of co-culture of PBMC and tumor cell lines with 1 nM of the different TROP-2 bsAbs. Both CD4^+^ and CD8^+^ T cell numbers increased in response to all TROP-2 bsAbs, with no differences observed for differing CD3 affinities and TROP-2 binders with MDA-MB-231 as target cells. In the presence of RT4 cells, CD3low constructs showed a slightly higher degree of T cell expansion (**Figure 4A**). All TROP-2 bsAb constructs promoted the expansion of both central and effector memory T cell subsets, whereas no expansion was observed in the presence of isotype controls (**Figures 4B, C**). No significant differences were detected regarding the TROP-2 binders with respect to T cell expansion (**Supplementary Figures 6A, B**). T cell expansion was comparable across high and low TROP-2-expressing cell lines, indicating that long-term T cell stimulation does not require pronounced antigen density. Next, we examined long-term tumor control using an xCelligence real-time monitoring assay over 7 days. All TROP-2 bsAbs effectively suppressed tumor cell growth, with MDA-MB-231 cells requiring a longer period for complete clearance than RT4 cells (**Figure 4D**). Thus, all TROP-2×CD3 bsAbs - including those with low CD3 affinity and accordingly reduced cytokine induction - not only activate and engage T cells but also support durable T cell proliferation and tumor control, even in models with low TROP-2 expression.

## 4 Discussion

Despite substantial progress in solid tumor therapy over recent decades, effective treatment options for both TNBC and bladder cancer remain limited. Treatment of TNBC, unlike other breast cancer subtypes, is not susceptible to hormone-based therapeutics. The introduction of ICIs, most notably neoadjuvant pembrolizumab, represents a significant advance in TNBC management, leading to improved pathologic complete response rates and establishing the clinical relevance of T cell-based therapies in this disease (23, 24). However, only about 5-20% of patients ultimately benefit from ICI treatment, and responses remain highly dependent on the molecular phenotype (25). Likewise, PARP inhibitors benefit only the small TNBC subset with BRCA alterations (26). More recently, ADCs have emerged as promising therapeutic approach, with SG approved for specific TNBC indications based on clinical criteria (26). Similarly, treatment options for bladder cancer expanded with the introduction of ICIs, small-molecule inhibitors, and ADCs (27), although the overall clinical benefit remains rather modest. Importantly, so far, no T cell-redirecting therapies, such as CAR-T cells or bsAbs, have been approved for the treatment of either TNBC or bladder cancer.

ADCs have emerged as a promising strategy in oncology by combining a monoclonal antibody targeting a TAA to deliver cytotoxic payloads selectively to tumor cells. However, their clinical efficacy is often constrained by factors such as heterogeneous TAA expression, dependence on receptor internalization for payload release, and systemic off-target toxicities (28, 29). Importantly, many ADCs require high antigen density, often estimated to be above ~10, 000 copies/cell, to achieve meaningful efficacy, although this threshold varies with internalization kinetics, epitope accessibility, and payload potency (30, 31). Like ADCs, bsAbs rely on sufficient expression of the TAA for their selective activity. However, the CD3-binding arm enables potent T cell-redirection and -mediated cytotoxicity, reducing the dependence on high antigen density and allowing effective activity in tumors with heterogeneous or low antigen expression.

TROP-2 has been widely explored as an attractive therapeutic target due to its elevated expression across multiple solid cancer types, including TNBC and bladder cancer, and this is in line with the data obtained in our study using TCGA datasets (13, 32). TNBC and bladder cancer represent clinically distinct epithelial malignancies that nonetheless share key challenges, including limited responsiveness to current immunotherapies and heterogeneous TROP-2 expression. As of today, two TROP-2-targeting ADCs (SG and Dato-DXd) have received clinical approval. While SG has demonstrated benefit in TNBC (NCT02574455) (33), clinical evaluation in bladder cancer failed to improve overall survival (NCT04527991) (34), a limitation rather driven by early toxicity than insufficient TROP-2 expression. These observations point to a better suitability of TROP-2-directed strategies that do not depend on payload delivery, such as bsAbs.

Here, we designed TROP-2×CD3 bsAbs incorporating the antibody clones RS7 and TINA1, derived from the clinically validated TROP-2-targeted ADCs SG and Dato-DXd. The selection of these TROP-2 binding domains was guided by their well-characterized specificity and affinity, as well as their prior clinical development, which provides an established framework regarding target accessibility and safety in patients. Leveraging binders with demonstrated clinical performance was intended to enhance the translational relevance of the resulting bispecific constructs. These bsAbs were subsequently evaluated in TNBC and bladder cancer models spanning high and low TROP-2 expression to assess robustness across heterogenous antigen levels. With all constructs, we observed induction of robust T cell activation, cytokine release, and potent tumor cell killing in both short- and long-term assays. Importantly, all TROP-2 bsAbs in our panel promoted sustained T cell expansion and memory formation, a key requirement to combat high tumor burden and for durable anti-tumor immunity (35, 36). Notably, with both TROP-2 binders, activity was maintained even with low TAA expression levels, as revealed by analyses with the TROP-2 low-expressing MDA-MB-231 cells, whereas no T cell activation or cytotoxicity was observed in the TROP-2-negative PANC-1 cell line. These findings support the antigen specificity of our TROP-2×CD3 bsAbs. However, we acknowledge that the absence of isogenic TROP-2 knockdown or knockout models limits the ability to definitively attribute all observed effects exclusively to TROP-2-dependent mechanisms. Future studies employing CRISPR/Cas9-mediated knockout or inducible knockdown systems in otherwise identical cellular backgrounds would provide more rigorous mechanistic validation of TROP-2-specific activity. In line with potency, RS7- and TINA1-based bsAbs exhibited EC_50_ values for cytotoxicity in the picomolar range (~0.006 nM for CD3high and ~0.015 nM for CD3low) for MDA-MB-231 cells, while SG displayed poorly activity in the same model (37). Of note, Goldenberg et al. showed that SG failed to achieve effective tumor reduction in MDA-MB-231 models (38), whereas Cardillo et al. observed that TROP-2 high-expressing MDA-MB-231 cells were significantly more susceptible to this ADC (37). Similarly, Li et al. reported that Dato-DXd was ineffective in reducing tumor burden in MDA-MB-231 models at lower doses (39). In this context, our findings highlight a key advantage of T cell-engaging bsAbs over ADCs: bsAbs can mediate potent tumor cell killing even in settings with low TAA expression, thereby overcoming the dependence on high target density and antigen internalization that constrains the efficacy of ADCs.

Interestingly, although RS7- and TINA1-based antibodies are known to undergo internalization in TROP-2–expressing cells, we observed robust tumor cell killing in our assays. Consistent with this, our 24 h binding analyses showed preserved TROP-2 surface availability, indicating that internalization does not abrogate epitope accessibility. In agreement with these findings, Sharkey et al. (40) reported that hRS7 IgG internalizes approximately 32 ± 2.09% after 24 h, and that an RS7-based Tri-Fab bsAb (TF12) internalizes about 40% after over the same period, while remaining detectable on the cell membrane. These data suggest that a sufficient fraction of TROP-2 persists at the surface to support continued bsAb binding and T cell-mediated cytotoxicity. Moreover, we demonstrate that our TROP-2×CD3 bsAbs do not affect tumor cell proliferation or migration in the absence of T cells, regardless of TROP-2 expression level. This is consistent with previous reports using hRS7 IgG (41) and supports the conclusion that these constructs do not directly modulate TROP-2-driven tumor biology but act strictly as conditional T cell engagers.

Beyond target antigen density, we also investigated how selection of the TROP-2 binder (TINA-1 vs. RS7) and the CD3 binder (high- vs. low-affinity) influenced bsAb activity. CD3 affinity was tuned based on established principles in T cell-engaging antibody engineering, aiming to achieve a balance between potent tumor cell killing and controlled T cell activation, thereby minimizing the risk of systemic immune activation (42, 43). In our IgGsc bsAb format, RS7- and TINA-1-based constructs displayed no measurable differences in tumor cell binding, T cell activation, tumor cell killing, or T cell proliferative capacity. These findings are consistent with recent reports showing that Dato-DXd (clone TINA1) and SG (clone RS7) recognize overlapping epitopes on TROP-2 (44), which likely explains the absence of functional differences between the two TROP-2 binders in our study. Although Dato-DXd and SG were reported to exhibit affinities in the nanomolar range with notable differences in KD and Kd (44), these disparities did not translate into differential activity within our bsAb format. Furthermore, both TROP-2 binders retained comparable potency when paired with either CD3high or CD3low constructs, despite reduced binding of CD3low variants to both CD4^+^ and CD8^+^ T cells. This is of importance, as CD3low configurations consistently induced lower IFN-γ and IL-2 secretion compared to CD3high constructs. In line with previous studies (20, 43), these findings indicate that certain TAA binders can be combined with a CD3low binder without compromising antitumor efficacy, while potentially mitigating cytokine-release and thus potentially dose-limiting toxicities.

Although TROP-2 expression in normal tissues is generally limited, its presence in certain epithelial compartments (10) requires careful on-target, off-tumor safety considerations for T cell-redirecting approaches, particularly in organs such as lung and kidney. While TROP-2-targeting ADCs have shown manageable safety profiles in clinical settings, Sun et al., reported that TROP-2 targeted CAR-T cells can induce inflammation-mediated toxicity in TROP-2-positive tissues in humanized mice (45), underscoring the potential risk associated with potent T cell engagement. In this context, the reduced IFN-γ and IL-2 secretion and slower activation kinetics observed with CD3low constructs suggest a more controlled T cell response, which may mitigate the risk of cytokine release syndrome. Importantly, CD3 affinity modulation also increased the activation threshold of T cells, resulting in greater dependency on antigen expression levels. This was reflected in MCF10A cells, where CD3low constructs, and notably TINA×CD3low, required higher concentrations to induce cytotoxicity and cytokine release, while maintaining robust activity in tumor cell lines. Furthermore, TINA-based bsAbs demonstrated a higher selectivity index in TROP-2 high expressing tumor cells (>50), compared with RS7-based constructs (~20), indicating that binder selection can further enhance tumor selectivity. Together, these findings support the concept that CD3 affinity tuning combined with an optimized TROP-2 targeting can broaden the therapeutic window of TROP-2×CD3 bsAbs, preserving anti-tumor potency while potentially reducing off-tumor effects.

A number of therapeutics strategies targeting TROP-2 are currently being explored in clinical trials, highlighting the growing interest in this antigen as a target. Several programs are ongoing, including expanded indications for SG and Dato-DXd (NCT07077564, NCT06235216, NCT06865677, NCT06176261, NCT07129993), as well as the evaluation of novel Trop-2-targeting ADCs (NCT06793332, NCT07067567). Other clinical trials are evaluating TROP-2 as a target for adoptive cellular therapies, including CAR-T/-NK cells (NCT06066424, NCT06454890), but to date no clinical study investigates TROP-2-directed bsAbs. While ICIs, such as pembrolizumab, have established the clinical relevance of T cell-based therapies in TNBC, their efficacy depends on pre-existing antitumor immunity and thus is limited to a subset of patients (23, 24). In contrast, T cell-engaging bsAbs actively recruit and redirect T cells toward tumor cells independently of baseline immune infiltration. This suggests that TROP-2×CD3 bsAbs may offer therapeutic benefit in ICI-refractory or immunologically “cold” tumors and could potentially be explored in combination with or sequentially to checkpoint blockade to broaden patient benefit.

A limitation of the present study is that all functional analyses were performed *in vitro* and therefore do not fully recapitulate the complexity of the tumor microenvironment, including immunosuppressive cell populations and stromal barriers. Importantly, *in vitro* systems cannot adequately predict pharmacokinetics, biodistribution, systemic immune activation, or organ specific toxicities that may emerge *in vivo* following administration of T cell-redirecting bsAbs. In particular, the risk of cytokine release syndrome and associated hepatic or renal toxicities (46) must be carefully evaluated in appropriate animal models. Furthermore, given the physiological expression of TROP-2 in certain epithelial compartments, *in vivo* studies are essential to assess the potential for on-target, off-tumor toxicity and to define the therapeutic window of TROP-2-targeted bsAbs. To address these considerations, evaluation in relevant preclinical models will be required. Because both RS7- and TINA1-derived constructs recognize human TROP-2, translational assessment will necessitate either TROP-2-humanized mouse models, or non-human primates, such as cynomolgus monkeys, which more closely recapitulate human TROP-2 distribution (47). Although these studies were beyond the scope of the present work, they represent critical next steps toward clinical translation of this therapeutic strategy.

In summary, our data show that leveraging TROP-2 as a target for bsAbs enables potent cytotoxicity and sustained T cell activation even in tumors with low or heterogeneous antigen expression. Moreover, the choice of TROP-2 binder influences tumor selectivity, with TINA-based constructs exhibiting higher selectivity for cells with elevated TROP-2 expression compared with RS7, highlighting the potential to further optimize the therapeutic window in combination with reduced CD3 affinity. These findings provide a rationale for the dual tuning of TROP-2 and CD3 binders and support further preclinical evaluation of TROP-2×CD3low bsAbs as promising immunotherapeutic candidates and warrant exploration in *in vivo* models ultimately required evaluation in clinical studies.

## Data Availability

The raw data supporting the conclusions of this article will be made available by the authors upon request. No public repository deposition was performed, and therefore no accession number applies.

## References

[1] Ribas A Wolchok JD . Cancer immunotherapy using checkpoint blockade. Science. (2018) 359:1350–5. doi: 10.1126/science.aar4060, PMID: 29567705 PMC7391259

[2] Daver N Alotaibi AS Bucklein V Subklewe M . T-cell-based immunotherapy of acute myeloid leukemia: current concepts and future developments. Leukemia. (2021) 35:1843–63. doi: 10.1038/s41375-021-01253-x, PMID: 33953290 PMC8257483

[3] Brudno JN Maus MV Hinrichs CS . CAR T cells and T-cell therapies for cancer: A translational science review. JAMA. (2024) 332:1924–35. doi: 10.1001/jama.2024.19462, PMID: 39495525 PMC11808657

[4] Wang Z Xie Y Wang JQ Cheng Y Fleishman J Chen ZS . Tebentafusp: a novel drug for the treatment of metastatic uveal melanoma. Drugs Today (Barc). (2023) 59:179–93. doi: 10.1358/dot.2023.59.3.3542417, PMID: 36847626

[5] Ahn MJ Cho BC Felip E Korantzis I Ohashi K Majem M . Tarlatamab for patients with previously treated small-cell lung cancer. N Engl J Med. (2023) 389:2063–75. doi: 10.1056/NEJMoa2307980, PMID: 37861218

[6] Galluzzi L Chan TA Kroemer G Wolchok JD Lopez-Soto A . The hallmarks of successful anticancer immunotherapy. Sci Transl Med. (2018) 10:eaat7807. doi: 10.1126/scitranslmed.aat7807, PMID: 30232229

[7] Baeuerle PA Wesche H . T-cell-engaging antibodies for the treatment of solid tumors: challenges and opportunities. Curr Opin Oncol. (2022) 34:552–8. doi: 10.1097/CCO.0000000000000869, PMID: 35880455 PMC9415207

[8] Qiu S Zhang J Wang Z Lan H Hou J Zhang N . Targeting Trop-2 in cancer: Recent research progress and clinical application. Biochim Biophys Acta Rev Cancer. (2023) 1878:188902. doi: 10.1016/j.bbcan.2023.188902, PMID: 37121444

[9] Trerotola M Cantanelli P Guerra E Tripaldi R Aloisi AL Bonasera V . Upregulation of Trop-2 quantitatively stimulates human cancer growth. Oncogene. (2013) 32:222–33. doi: 10.1038/onc.2012.36, PMID: 22349828

[10] Stepan LP Trueblood ES Hale K Babcook J Borges L Sutherland CL . Expression of Trop2 cell surface glycoprotein in normal and tumor tissues: potential implications as a cancer therapeutic target. J Histochem Cytochem. (2011) 59:701–10. doi: 10.1369/0022155411410430, PMID: 21551320 PMC3201164

[11] Lin H Huang JF Qiu JR Zhang HL Tang XJ Li H . Significantly upregulated TACSTD2 and Cyclin D1 correlate with poor prognosis of invasive ductal breast cancer. Exp Mol Pathol. (2013) 94:73–8. doi: 10.1016/j.yexmp.2012.08.004, PMID: 23031786

[12] Fong D Moser P Krammel C Gostner JM Margreiter R Mitterer M . High expression of TROP2 correlates with poor prognosis in pancreatic cancer. Br J Cancer. (2008) 99:1290–5. doi: 10.1038/sj.bjc.6604677, PMID: 18813308 PMC2570520

[13] Liao Q Zhang R Ou Z Ye Y Zeng Q Wang Y . TROP2 is highly expressed in triple-negative breast cancer CTCs and is a potential marker for epithelial mesenchymal CTCs. Mol Ther Oncol. (2024) 32:200762. doi: 10.1016/j.omton.2024.200762, PMID: 38596285 PMC10869581

[14] Wahby S Fashoyin-Aje L Osgood CL Cheng J Fiero MH Zhang L . FDA approval summary: accelerated approval of sacituzumab govitecan-hziy for third-line treatment of metastatic triple-negative breast cancer. Clin Cancer Res. (2021) 27:1850–4. doi: 10.1158/1078-0432.CCR-20-3119, PMID: 33168656

[15] Royce M Shah M Zhang L Cheng J Bonner MK Pegues M . FDA approval summary: datopotamab deruxtecan-dlnk for treatment of patients with unresectable or metastatic, HR-positive, HER2-negative breast cancer. Clin Cancer Res. (2025) 31:4405–11. doi: 10.1158/1078-0432.CCR-25-1388, PMID: 40864501 PMC12393668

[16] Ahn MJ Tanaka K Paz-Ares L Cornelissen R Girard N Pons-Tostivint E . Datopotamab deruxtecan versus docetaxel for previously treated advanced or metastatic non-small cell lung cancer: the randomized, open-label phase III TROPION-lung01 study. J Clin Oncol. (2025) 43:260–72. doi: 10.1200/JCO-24-01544, PMID: 39250535 PMC11771353

[17] Zekri L Vogt F Osburg L Muller S Kauer J Manz T . An IgG-based bispecific antibody for improved dual targeting in PSMA-positive cancer. EMBO Mol Med. (2021) 13:e11902. doi: 10.15252/emmm.201911902, PMID: 33372710 PMC7863392

[18] Vidula N Yau C Rugo H . Trophoblast Cell Surface Antigen 2 gene (TACSTD2) expression in primary breast cancer. Breast Cancer Res Treat. (2022) 194:569–75. doi: 10.1007/s10549-022-06660-x, PMID: 35789445

[19] Aslan M Hsu EC Garcia-Marques FJ Bermudez A Liu S Shen M . Oncogene-mediated metabolic gene signature predicts breast cancer outcome. NPJ Breast Cancer. (2021) 7:141. doi: 10.1038/s41523-021-00341-6, PMID: 34711841 PMC8553750

[20] Zekri L Lutz M Prakash N Manz T Klimovich B Mueller S . An optimized IgG-based B7-H3xCD3 bispecific antibody for treatment of gastrointestinal cancers. Mol Ther. (2023) 31:1033–45. doi: 10.1016/j.ymthe.2023.02.010, PMID: 36793213 PMC10124076

[21] Shih LB Xuan H Aninipot R Stein R Goldenberg DM . *In vitro* and *in vivo* reactivity of an internalizing antibody, RS7, with human breast cancer. Cancer Res. (1995) 55:5857s–63s. 7493360

[22] Chang CH Gupta P Michel R Loo M Wang Y Cardillo TM . Ranpirnase (frog RNase) targeted with a humanized, internalizing, anti-Trop-2 antibody has potent cytotoxicity against diverse epithelial cancer cells. Mol Cancer Ther. (2010) 9:2276–86. doi: 10.1158/1535-7163.MCT-10-0338, PMID: 20663928

[23] Cortes J Rugo HS Cescon DW Im SA Yusof MM Gallardo C . Pembrolizumab plus chemotherapy in advanced triple-negative breast cancer. N Engl J Med. (2022) 387:217–26. doi: 10.1056/NEJMoa2202809, PMID: 35857659

[24] Schmid P Cortes J Dent R McArthur H Pusztai L Kummel S . Overall survival with pembrolizumab in early-stage triple-negative breast cancer. N Engl J Med. (2024) 391:1981–91. doi: 10.1056/NEJMoa2409932, PMID: 39282906

[25] Debien V De Caluwe A Wang X Piccart-Gebhart M Tuohy VK Romano E . Immunotherapy in breast cancer: an overview of current strategies and perspectives. NPJ Breast Cancer. (2023) 9:7. doi: 10.1038/s41523-023-00508-3, PMID: 36781869 PMC9925769

[26] Riaz F Gruber JJ Telli ML . New treatment approaches for triple-negative breast cancer. Am Soc Clin Oncol Educ Book. (2025) 45:e481154. doi: 10.1200/EDBK-25-481154, PMID: 40460322

[27] Kumbham S Md Mahabubur Rahman K Foster BA You Y . A comprehensive review of current approaches in bladder cancer treatment. ACS Pharmacol Transl Sci. (2025) 8:286–307. doi: 10.1021/acsptsci.4c00663, PMID: 39974639 PMC11833730

[28] Wang R Hu B Pan Z Mo C Zhao X Liu G . Antibody-Drug Conjugates (ADCs): current and future biopharmaceuticals. J Hematol Oncol. (2025) 18:51. doi: 10.1186/s13045-025-01704-3, PMID: 40307936 PMC12044742

[29] Zhou X Han Y Fang Y Ma P Zhou J Jiang Y . Antibody-drug conjugates: Current challenges and innovative solutions for precision cancer therapy. Med. (2025) 6:100849. doi: 10.1016/j.medj.2025.100849, PMID: 41033317

[30] Metrangolo V Engelholm LH . Antibody-drug conjugates: the dynamic evolution from conventional to next-generation constructs. Cancers (Basel). (2024) 16:447. doi: 10.3390/cancers16020447, PMID: 38275888 PMC10814585

[31] Drago JZ Modi S Chandarlapaty S . Unlocking the potential of antibody-drug conjugates for cancer therapy. Nat Rev Clin Oncol. (2021) 18:327–44. doi: 10.1038/s41571-021-00470-8, PMID: 33558752 PMC8287784

[32] Chou J Trepka K Sjostrom M Egusa EA Chu CE Zhu J . TROP2 expression across molecular subtypes of urothelial carcinoma and enfortumab vedotin-resistant cells. Eur Urol Oncol. (2022) 5:714–8. doi: 10.1016/j.euo.2021.11.005, PMID: 35216942 PMC10262920

[33] Bardia A Hurvitz SA Tolaney SM Loirat D Punie K Oliveira M . Sacituzumab govitecan in metastatic triple-negative breast cancer. N Engl J Med. (2021) 384:1529–41. doi: 10.1056/NEJMoa2028485, PMID: 33882206

[34] Powles T Tagawa S Vulsteke C Gross-Goupil M Park SH Necchi A . Sacituzumab govitecan in advanced urothelial carcinoma: TROPiCS-04, a phase III randomized trial. Ann Oncol. (2025) 36:561–71. doi: 10.1016/j.annonc.2025.01.011, PMID: 39934055

[35] Ando M Ito M Srirat T Kondo T Yoshimura A . Memory T cell, exhaustion, and tumor immunity. Immunol Med. (2020) 43:1–9. doi: 10.1080/25785826.2019.1698261, PMID: 31822213

[36] Klebanoff CA Gattinoni L Restifo NP . CD8+ T-cell memory in tumor immunology and immunotherapy. Immunol Rev. (2006) 211:214–24. doi: 10.1111/j.0105-2896.2006.00391.x, PMID: 16824130 PMC1501075

[37] Cardillo TM Rossi DL Zalath MB Liu D Arrojo R Sharkey RM . Predictive biomarkers for sacituzumab govitecan efficacy in Trop-2-expressing triple-negative breast cancer. Oncotarget. (2020) 11:3849–62. doi: 10.18632/oncotarget.27766, PMID: 33196706 PMC7597411

[38] Goldenberg DM Cardillo TM Govindan SV Rossi EA Sharkey RM . Trop-2 is a novel target for solid cancer therapy with sacituzumab govitecan (IMMU-132), an antibody-drug conjugate (ADC). Oncotarget. (2015) 6:22496–512. doi: 10.18632/oncotarget.4318, PMID: 26101915 PMC4673178

[39] Li WF Chiang MF Weng HC Yang JJ Wu HS Wu SY . OBI-992, a novel TROP2-targeted antibody-drug conjugate, demonstrates antitumor activity in multiple cancer models. Mol Cancer Ther. (2025) 24:163–75. doi: 10.1158/1535-7163.MCT-24-0588, PMID: 39786401 PMC11791482

[40] Sharkey RM van Rij CM Karacay H Rossi EA Frielink C Regino C . A new Tri-Fab bispecific antibody for pretargeting Trop-2-expressing epithelial cancers. J Nucl Med. (2012) 53:1625–32. doi: 10.2967/jnumed.112.104364, PMID: 22952342

[41] Deng J Geng Z Luan L Jiang D Lu J Zhang H . Novel anti-trop2 nanobodies disrupt receptor dimerization and inhibit tumor cell growth. Pharmaceutics. (2024) 16:1255. doi: 10.3390/pharmaceutics16101255, PMID: 39458590 PMC11510716

[42] Haber L Olson K Kelly MP Crawford A DiLillo DJ Tavare R . Generation of T-cell-redirecting bispecific antibodies with differentiated profiles of cytokine release and biodistribution by CD3 affinity tuning. Sci Rep. (2021) 11:14397. doi: 10.1038/s41598-021-93842-0, PMID: 34257348 PMC8277787

[43] Poussin M Sereno A Wu X Huang F Manro J Cao S . Dichotomous impact of affinity on the function of T cell engaging bispecific antibodies. J Immunother Cancer. (2021) 9:e002444. doi: 10.1136/jitc-2021-002444, PMID: 34253637 PMC8276301

[44] Chang TY Lin CJ Wen SN Wu YC Wei CY Huang JY . Preclinical evaluation of a novel antibody-drug conjugate OBI-992 for Cancer therapy. Sci Rep. (2025) 15:8735. doi: 10.1038/s41598-025-92697-z, PMID: 40082588 PMC11906863

[45] Sun S Wang X Chen Y Liang Z Nian Z Xu W . Preclinical evaluation of antitumor activity and toxicity of TROP2-specific CAR-T cells for treatment of triple-negative breast cancer. J Immunother Cancer. (2025) 13:e012442. doi: 10.1136/jitc-2025-012442, PMID: 40903191 PMC12410632

[46] Jain MD Abramson JS Ansell SM . Easy as ABC: managing toxicities of antibody-drug conjugates, bispecific antibodies, and CAR T-cell therapies. Am Soc Clin Oncol Educ Book. (2025) 45:e473916. doi: 10.1200/EDBK-25-473916, PMID: 40294348

[47] Cardillo TM Govindan SV Sharkey RM Trisal P Goldenberg DM . Humanized anti-Trop-2 IgG-SN-38 conjugate for effective treatment of diverse epithelial cancers: preclinical studies in human cancer xenograft models and monkeys. Clin Cancer Res. (2011) 17:3157–69. doi: 10.1158/1078-0432.CCR-10-2939, PMID: 21372224 PMC10766325

