# Supplementary material for: Characterization of TROP-2 bispecific T cell engagers for immunotherapy of triple negative breast and bladder cancer
Source: Front Immunol. 2026 Mar 12;17:1794705. doi: 10.3389/fimmu.2026.1794705 (PMC13017863; doi:10.3389/fimmu.2026.1794705)
Supplement: Supplementary file 1 [file DataSheet1.docx]

Supplementary Material

# Supplementary Figures and Tables

## Supplementary Figure 1

**B**

**A**

**C**

**D**

**E**

TINA1×CD3*high*

TINA1×CD3*low*

RS7×CD3*low*

RS7×CD3*high*

MOPC×CD3*high*

MOPC×CD3*low*

Untreated

**Supplementary Figure 1. Characterization of TROP-2 expression, internalization, and antibody-mediated effects on proliferation and migration. (A)** TROP-2 molecules per cell were quantified in bladder cancer (RT4 and T-24) and TNBC (HCC70 and MDA-MB-231) cell lines using flow cytometry. **(B)** RT4 and MDA-MB-231 cell lines were incubated for 24h with maximal concentration of TROP-2xCD3high bsAbs. After 24h bsAb binding was assessed by flow cytometry. **(C–D)** Proliferation of RT4 and MDA-MB-231 cells in the presence of TROP-2×CD3 bsAbs (2 nM), assessed by **(C)** WST staining and **(D)** CellTiter-Glo, shown as absorbance at 450–620 nm and relative luminescence units (RLU), respectively. **(E)** Relative wound density over time in RT4 and MDA-MB-231 cells, assessing the effect of TROP-2×CD3 bsAbs (10 nM) on cell migration. Mean ± standard error of the mean (SEM) is shown.

## Supplementary Figure 2

**RS7 x CD3*high***

**RS7 x CD3*low***

**TINA1 x CD3*low***

**TINA1 x CD3*high***


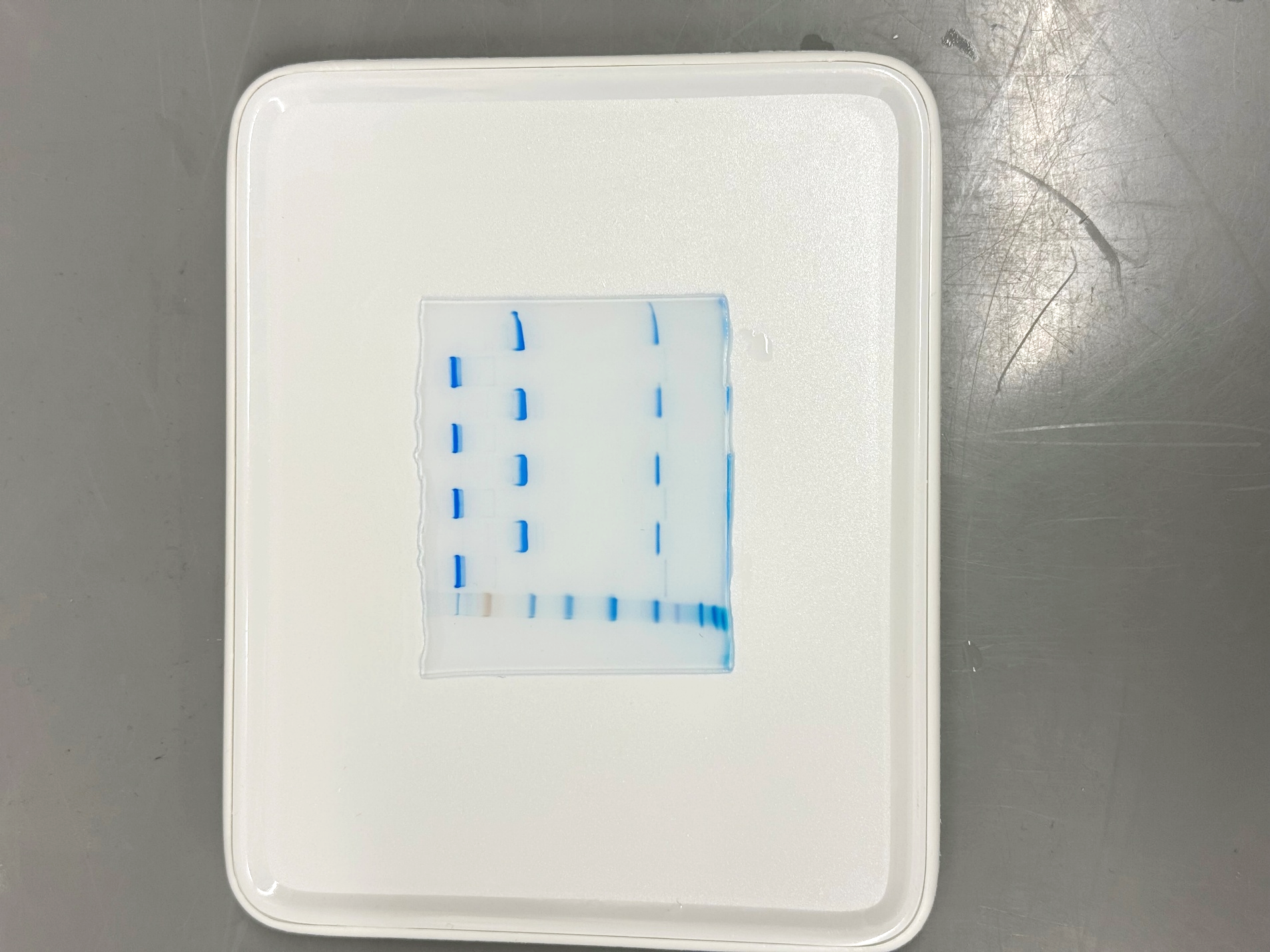


**kDa**

**R**

**R**

**R**

**NR**

**NR**

**R**

**NR**

**NR**

**98**

**198**

**62**

**38**

**49**

**28**

**14**

**17**

**6**

**Supplementary Figure 2. Purity and integrity of TROP-2 bsAbs.** Purified TINA-1×CD3*high*, TINA-1×CD3*low*, RS7×CD3*high*, and RS7×CD3*low* were analyzed by SDS-PAGE under non-reducing (NR) and reducing (R) conditions, followed by Coomassie G-250 staining.

##
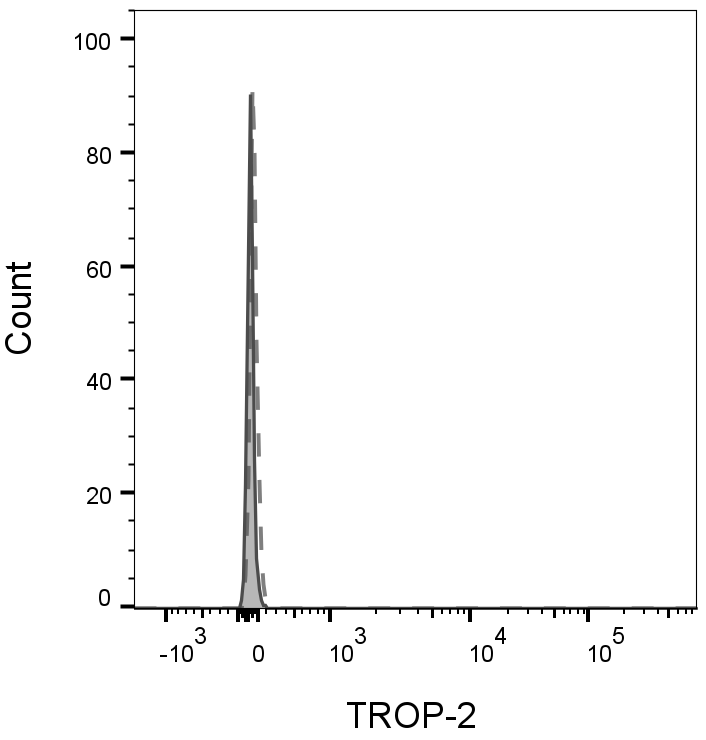
Supplementary Figure 3

**C**

**B**

**A**

**PANC-1**

**E**

**D**

**Supplementary Figure 3. Target-dependent activity of TROP-2 bsAbs. (A**) PANC-1 cells were analyzed for TROP-2 expression by flow cytometry and confirmed to be TROP-2-negative. **(B)** PANC-1 cells were incubated with titrated concentrations of TROP-2 bsAbs, binding was assessed by flow cytometry. No unspecific binding was detected. (**C-E**) T cell activation, cytotoxicity, and cytokine release were assessed in the PANC-1 cell line in co-culture with PBMCs (n=2) at an E:T ratio of 2:1. Data represent pooled results from PBMCs derived from two independent donors. (**C**) CD4⁺ and CD8⁺ T cell activation was assessed by flow cytometry based on CD25 expression after 72 hours. (**D**) Cytotoxicity against PANC-1 cells was evaluated after 72 h by flow cytometry, and the percentage of viable target cells is shown. (**E**) IFN-γ secretion was quantified 8 hours after the initiation of co-culture by ELISA. Mean ± standard error of the mean (SEM) is shown.

## Supplementary Figure 4

**A**

**B**

**Supplementary Figure 4. IL-6 and TNF levels following TROP-2×CD3 bsAbs stimulatioin. (A)** IL-6 and **(B)** TNF levels were quantified by LEGENDplex in supernatants after 8h from PBMC (n = 4) co-cultured with RT4 or MDA-MB-231 cells at an E:T ratio of 2:1. Cytokine secretion was assessed across a titration of TROP-2×CD3 bsAbs. Mean ± standard error of the mean (SEM) is shown.

##
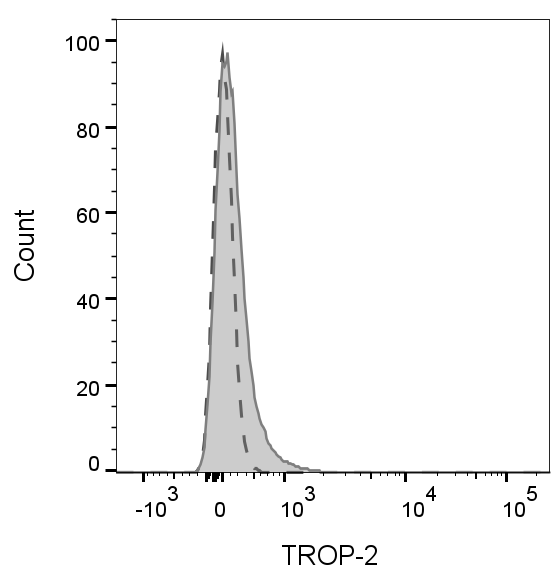
Supplementary Figure 5

**C**

**B**

**A**

**D**

**E**

**F**

**G**

| bsAb Constructs | RT4 normalized  EC_50_ [nM] | MDA-MB-231 normalized  EC_50_ [nM] | MCF10A normalized  EC_50_ [nM] | Selectivity index (MCF10A / RT4) | Selectivity index (MCF10A / MDA-MB-231) |
| --- | --- | --- | --- | --- | --- |
| TINA1×CD3*high* | 0,0034 | 0,0127 | 0,1778 | 52x | 14x |
| TINA1×CD3*low* | 0,0081 | 0,0299 | 0,5021 | 62x | 17x |
| RS7×CD3*high* | 0,0059 | 0,0086 | 0,1066 | 18x | 12x |
| RS7×CD3*low* | 0,0099 | 0,0089 | 0,1465 | 15x | 16x |

**Supplementary Figure 5. Activity of TROP-2×CD3 bsAbs in TROP-2 very low-expressing MCF10A cell line. (A)** Surface TROP-2 expression in MCF10A cells analyzed by flow cytometry. **(B)** TROP-2 molecules per cell in and expressed relative to RT4 cells. **(C)** Binding of titrated TROP-2×CD3 bsAbs to MCF10A cells assessed by flow cytometry. **(D–F)** Functional activity in co-cultures of MCF10A cells with PBMCs (n=2) at an E:T ratio of 2:1. **(D)** CD4^⁺^ and CD8^⁺^ T cell activation measured by CD25 expression after 72 h. **(E)** Cytotoxicity determined after 72 h and expressed as percentage of viable MCF10A cells. **(F)** IFN-γ secretion quantified by ELISA 8 h after co-culture initiation**. (G)** Summary table of cytotoxic EC_50_ values in RT4, MDA-MB-231, and MCF10A cells, including calculated selectivity indices (MCF10A vs RT4 and MCF10A vs MDA-MB-231). Mean ± standard error of the mean (SEM) is shown.

## Supplementary Figure 6

**A**

**B**

**Supplementary Figure 6. T cell memory subset total count**. RT4 and MDA-MB-231 cell lines were co-cultured with PBMCs (n=4) and the indicated bsAbs (1 nM) for 6 days at an E:T ratio of 2:1. Tumor cells and bsAbs were renewed on day 3. CD4^+^ and CD8^+^ T cell subsets were evaluated by flow cytometry on day 6. Central memory (CD62L^+^CD45RO^+^) and effector memory (CD62L^-^ CD45RO^+^) T cell counts with **(A)** RT4 and **(B)** MDA-MB-231 are shown. Mean ± standard error of the mean (SEM) is shown. Statistical significance was calculated using one-way ANOVA with Bonferroni correction for multiple comparisons. *p < 0.05, **p < 0.01, ***p < 0.001, ****p < 0.0001.
